# Prepregnancy body mass index and gestational weight gain are associated with maternal and infant adverse outcomes in Chinese women with gestational diabetes

**DOI:** 10.1038/s41598-022-06733-3

**Published:** 2022-02-17

**Authors:** Qing-Xiang Zheng, Hai-Wei Wang, Xiu-Min Jiang, Yan Lin, Gui-Hua Liu, Mian Pan, Li Ge, Xiao-Qian Chen, Jing-Ling Wu, Xiao-Yun Zhang, Yu-Qing Pan, Hong-Gu He

**Affiliations:** 1 Fujian Maternity and Child Health Hospital, Affiliated Hospital of Fujian Medical University, Fuzhou, China; 2 School of Nursing, Fujian University of Traditional Chinese Medicine, Fuzhou, China; 3 Alice Lee Centre for Nursing Studies, Yong Loo Lin School of Medicine, National University of Singapore, Level 2, Clinical Research Centre, 10 Medical Drive, Singapore, 117597 Singapore; 4 National University Health System, Singapore, Singapore; 5 Nursing Department, Fujian Maternity and Child Health Hospital, 18 Daoshan Road, Fuzhou, Fujian China

**Keywords:** Diseases, Health care, Risk factors

## Abstract

The gestational weight gain (GWG) range of Chinese women with gestational diabetes mellitus (GDM) remains unclear. Our objective was to identify the ranges of GWG in Chinese women with GDM and to investigate the associations between prepregnancy body mass index (BMI), GWG and maternal-infant adverse outcomes. Cases of GDM women who delivered singletons from 2013 to 2018 in a public hospital were collected. Logistic regression analysis was used to assess the joint effects of prepregnancy BMI and GWG on maternal-infant adverse outcomes. Ultimately, 14,578 women were collected. The ranges of GWG in Chinese women with GDM were different from the National Academy of Medicine’s (NAM) recommendation. The ranges of GWG of Chinese women with GDM in the underweight, normal weight, overweight and obese groups were 5.95–21.95 kg, 4.23–21.83 kg, 0.88–21.12 kg and − 1.76 to 19.95 kg, respectively. The risks of large for gestational age (LGA), macrosomia and caesarean delivery were significantly increased with the increasing prepregnancy BMI. Furthermore, the risks of LGA, macrosomia and caesarean delivery were significantly higher in the normal weight group with a GWG higher than the NAM recommendation. Similarly, in the overweight group with a GWG higher than the NAM recommendation, the risks of LGA were significantly higher, while the risks of macrosomia were significantly lower. Overall, we determined the range of GWG in different prepregnancy BMI groups. And GDM women with high prepregnancy BMI and excessive GWG were associated with the higher risks of maternal-infants adverse outcomes in China.

## Introduction

Gestational diabetes mellitus (GDM) is the most common pregnancy complication, threatening the health of pregnant women and their offspring^1,2^. The incidence of GDM is increasing^3^. A total of 14.8% of pregnant women in China currently are suffering from GDM^4^. GDM is associated with increased risks of maternal-infant adverse outcomes^5^. Women with GDM have a higher incidence of gestational hypertension, foetal growth restriction, premature delivery, caesarean delivery, postpartum haemorrhage, hyperinsulinaemia and hypoglycaemia^6^. Moreover, women with GDM are 7.5 times more likely to develop type 2 diabetes mellitus (T2DM) than women without GDM^7^, and their offspring are also at higher risks of developing childhood obesity^8^ and T2DM^9^. The development of GDM is associated with multiple factors. Women with a higher body mass index (BMI) before conception have a 4–9 times higher incidence of GDM than normal weight women^10^. Maternal obesity and a higher gestational weight gain (GWG) are associated with a higher risk of GDM^11,12^.

GWG represents the nutritional status of a pregnant woman during pregnancy. It is also an indicator of maternal fat accumulation and the growth of the uterus, placenta and foetus^13^. Prepregnancy BMI reflects maternal nutritional conditions before conception^3^. Abnormal GWG and prepregnancy BMI are both associated with the pregnancy complications and maternal-infant adverse outcomes^14,15^. A systematic review of 196,670 pregnant women found that 47% of women had a GWG greater than the National Academy of Medicine’s (NAM) recommended criteria, while 23% of women had a GWG below the recommended values. Women with a GWG higher than the recommended ranges were associated with a higher risk of adverse maternal-infant outcomes than those with a GWG within the recommended range^15^. However, due to differences in race, dietary habits and culture, the recommended ranges of NAM are not suitable for Chinese women^16^. The appropriate ranges of GWG in women with GDM are not clear in China. In this study, we aimed to identify the ranges of GWG for Chinese women with GDM and to investigate the associations of GWG and maternal-infant adverse outcomes in GDM women.

## Results

### Characteristics of the GDM patients

Totally, 14,578 Chinese women with GDM collected. The demographic and clinical characteristics of participants were showed in Table 1. According to the NAM guidelines, all women were divided into four groups based on the prepregnancy BMI. Seventy-three percent (10,623 of 14,578) of women had a normal BMI, while only 230 (1.6%) women were in the obese group (Table 1). There were 2013 women in the underweight group and 1694 women in the overweight group (Table 1). Furthermore, the gestational BMI gain was significantly different among the four groups. The gestational BMI was mostly increased in women in the underweight group (3.87 ± 2.09). In contrast, women in the obese group had a relatively lower increase of gestational BMI (2.56 ± 2.35). The gestational BMI gain in the normal weight group and overweight group was 3.68 ± 2.02 and 2.91 ± 2.07, respectively (Table 1).

**Table 1:** The demographic and clinical characteristics of participants (n = 14,578).

Variables	Underweight group (n = 2,031, BMI < 18.5)	Normal weight group (n = 10,623, 18.5 ≤ BMI < 25.0)	Overweight group (n = 1,694, 25.0 ≤ BMI < 30.0)	Obese group (n = 230, BMI ≥ 30.0)
Age (year)	29.03 ± 4.26	31.21 ± 4.65	32.25 ± 4.61	31.04 ± 4.90
Pre-pregnancy BMI (kg/m^2^)	17.53 ± 0.80	21.40 ± 1.71	26.67 ± 1.27	32.10 ± 2.24
Pre-pregnancy weight (kg)	44.98 ± 3.35	54.47 ± 5.44	67.78 ± 5.38	81.98 ± 7.74
BMI before delivery (kg/m^2^)	22.99 ± 1.77	26.54 ± 2.21	30.82 ± 2.19	35.63 ± 3.00
Weight before delivery (kg)	58.97 ± 5.43	67.54 ± 6.80	78.30 ± 7.34	91.05 ± 9.58
Gestational BMI gain (kg/m^2^)	3.87 ± 2.09	3.68 ± 2.02	2.91 ± 2.07	2.56 ± 2.35
Gestational weeks (week)	38.46 ± 2.31	38.43 ± 2.33	38.36 ± 2.32	38.45 ± 2.52
**Model of delivery [NO.(%)]**				
Vaginal birth	1528(75.61)	6715(63.21)	887(52.36)	101(43.91)
Cesarean delivery	499(24.69)	3876(36.49)	804(46.46)	127(55.22)
Induced labor or Abortion	4(0.20)	32(0.30)	3(0.18)	2(0.87)
**Type of infant [NO.(%)]**				
Classification by gestational age				
Premature infant	222(10.98)	1207(11.36)	234(13.81)	29(12.61)
Term infant	1805(89.31)	9389(88.38)	1457(86.00)	199(86.52)
Classification by birth weight				
Macrosomia	39(1.93)	552(5.20)	151(8.91)	30(13.04)
Classification by the association between gestational age and birth weight				
AGA	1728(85.50)	8907(83.85)	1335(78.81)	166(72.17)
LGA	80(3.96)	1020(9.60)	271(16.00)	53(23.04)
SGA	214(10.59)	644(6.06)	82(4.84)	9(3.91)
Occupation				
Self-employed	35(1.72)	244(2.30)	33(1.95)	4(1.74)
Public officer	43(2.12)	238(2.24)	36(2.13)	1(0.43)
Housewife	841(41.41)	4417(41.58)	729(43.03)	94(40.87)
Student	2(0.10)	1(0.01)	1(0.06)	0(0.00)
Medical personnel	19(0.94)	96(0.90)	4(0.24)	4(1.74)
Employee	956(47.07)	4856(45.71)	744(43.92)	114(49.57)
Freelance	139(6.84)	771(7.26)	147(8.68)	13(5.65)

*BMI* body mass index, *GWG* Gestational weight gain, *AGA* appositeness for gestational age, *LGA* large for gestational age, *SGA* small for gestational age.

### The ranges and rates of GWG in Chinese women with GDM are different from the NAM recommended values

We then calculated the GWG in GDM women in the underweight, normal weight, overweight and obese groups. The gestational weight was mostly increased in women in the underweight group (14 ± 4.06). In contrast, women in the obese group had a relatively low increase in gestational weight (9.1 ± 5.54) (Table 2). The GWG in the normal weight and overweight groups was 13.08 ± 4.47 and 10.54 ± 4.91, respectively (Table 2).

**Table 2:** Comparisons of the GWG in Chinese women with GDM with the NAM recommendation (n = 14,578).

Pre-pregnant BMI (kg/m^2^)	GWG (kg) $\overline{X}$ ± SD	The GWG range (kg) (minimum–maximum)		The rate of GWG (kg/week) [Mean(minimum–maximum)]			
		GDM women	The NAM recommended range *	GDM women	The NAM recommended range	The *t-value* of the rate of GDM women versus those of the NAM recommendation	*p*-value
Underweight group	14.00 ± 4.06	5.95–21.95	12.5–18	0.51(0.22–0.80)	0.51(0.44–0.58)	0.18	0.86
Normal weight group	13.08 ± 4.47	4.23–21.83	11.5–16	0.48(0.15–0.80)	0.42(0.35–0.50)	36.15	< 0.05*
Overweight group	10.54 ± 4.91	0.88–21.12	7–11.5	0.39(0.03–0.75)	0.28(0.23–0.33)	24.15	< 0.05*
Obese group	9.10 ± 5.54	− 1.76 to 19.95	5–9	0.33(− 0.09 to 0.76)	0.22(0.17–0.27)	8.24	< 0.05*

*BMI* body mass index, *GWG* Gestational weight gain, *GDM* gestational diabetes mellitus.

*The values were recommended by the National Academy of Medicine guidelines in 2009.

**Figure 1:**
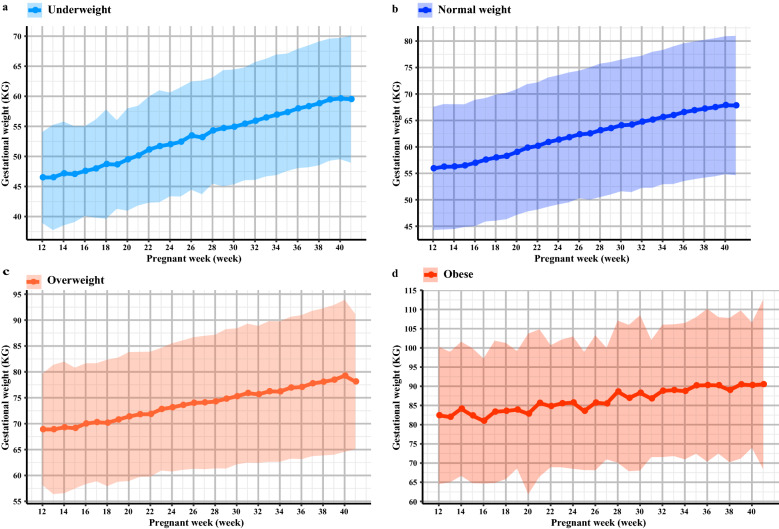
The GWG trajectory among Chinese women with GDM. (**a–d**) The GWG trajectory from 12th gestational week to 40th gestational week in underweight, normal weight, overweight and obesity group were shown, respectively.

The GWG ranges and GWG rates in GDM women in different prepregnancy BMI subgroups were also tested. We found that Chinese women with GDM had different GWG ranges in the underweight, normal weight, overweight and obese groups. The underweight GDM group had the narrowest GWG ranges (from 5.95 to 21.95 kg) (Table 2). Moreover, women in the underweight group had the fastest rate of GWG than the other groups (Fig. 1). The ranges of GWG in the normal weight and overweight groups were from 4.23 to 21.83 kg and from 0.88 to 21.12 kg, respectively. In contrast, GWG in the obese group had the widest GWG ranges (from − 1.76 to 19.95 kg) compared with the normal weight, overweight and obese groups. Additionally, the obese group had the slowest and fluctuating GWG rates (Fig. 1).

Previously, the GWG ranges and GWG rates in the four different BMI groups were tested by NAM. Therefore, we compared the GWG ranges and rates of Chinese women with GDM with the NAM recommendation. We found that in all underweight, normal weight, overweight and obese groups, the GWG ranges in Chinese women with GDM were significantly different from the criteria recommended by NAM (Table 2). Additionally, except for the underweight group, the rates of GWG among Chinese women with GDM were also different from those recommended by NAM (Table 2).

### Characteristics of the maternal-infant adverse outcomes of GDM patients

Women with GDM suffered from many maternal-infant adverse outcomes, including LGA, SGA, prematurity, macrosomia and caesarean delivery. We found that 10,112 of 14,578 GDM women suffered at least one of the adverse outcomes. The highest risk for GDM women was caesarean delivery. More than 36% of GDM women suffered from caesarean delivery, followed by LGA, prematurity and macrosomia adverse outcomes (Table 3).

**Table 3:** The risks of maternal or infant adverse outcomes of participants (n = 14,578).

Variables	n	n (%)					
		LGA	SGA	Prematurity	Macrosomia	Cesarean delivery	Total adverse outcomes
**Underweight group (Pre-pregnant BMI < 18.5, 12.5 kg ≤ Recommended GWG ≤ 18 kg)**	2031	80(3.94)	214(10.54)	217(10.68)	39(1.92)	499(24.57)	1049(51.65)
Lower than recommended GWG (GWG < 12.5 kg)	742	13(1.75)	100(13.48)	114(15.36)	7(0.94)	172(23.18)	406(54.72)
Equal to recommended GWG (12.5 kg ≤ GWG ≤ 18.0 kg)	997	42(4.21)	94(9.42)	79(7.92)	19(1.91)	233(23.37)	467(46.84)
Higher than recommended GWG (GWG > 18.0 kg)	292	25(8.56)	20(6.85)	24(8.22)	13(4.45)	94(32.19)	176(60.27)
**Normal weight group (18.5 ≤ Pre-pregnant BMI < 25.0, 11.5 kg ≤ Recommended GWG ≤ 16 kg)**	10,623	1020(9.60)	644(6.06)	1182(11.13)	552(5.20)	3876(36.49)	7274(68.47)
Lower than recommended GWG (GWG < 11.5 kg)	3894	214(5.50)	273(7.01)	527(13.53)	111(2.85)	1249(32.07)	2374(60.97)
Equal to recommended GWG (11.5 kg ≤ GWG ≤ 16.0 kg)	4303	436(10.13)	253(5.88)	397(9.23)	223(5.18)	1579(36.70)	2888(67.12)
Higher than recommended GWG (GWG > 16.0 kg)	2426	370(15.25)	118(4.86)	258(10.63)	218(8.99)	1048(43.20)	2012(82.93)
**Overweight group (25.0 ≤ Pre-pregnant BMI < 30.0, 7 kg ≤ Recommended GWG ≤ 11.5 kg)**	1694	271(15.98)	82(4.84)	233(13.75)	151(8.91)	804(47.46)	1541(90.97)
Lower than recommended GWG (GWG < 7 kg)	396	32(8.08)	24(6.06)	61(15.40)	16(4.04)	159(40.15)	292(73.74)
Equal to recommended GWG (7 kg ≤ GWG ≤ 11.5 kg)	629	88(13.99)	32(5.09)	92(14.63)	44(7.00)	291(46.26)	547(86.96)
Higher than recommended GWG (GWG > 11.5 kg)	669	151(22.57)	26(3.89)	80(11.96)	91(13.60)	354(52.91)	702(104.93)
**Obese group (Pre-pregnant BMI ≥ 30.0, 5 kg ≤ Recommended GWG ≤ 9 kg)**	230	53(21.14)	9(3.91)	29(12.61)	30(13.04)	127(55.22)	248(107.83)
Lower than recommended GWG (GWG < 5 kg)	50	8(16.00)	3(6.00)	8(16.00)	4(8.00)	22(44)	45(90.00)
Equal to recommended GWG (5 kg ≤ GWG ≤ 9 kg)	72	13(18.06)	6(8.33)	11(15.27)	7(9.72)	41(56.94)	78(108.33)
Higher than recommended GWG (GWG > 9 kg)	108	32(29.63)	0	10(9.26)	19(17.59)	64(59.26)	125(115.74)

*BMI* body mass index, *GWG* Gestational weight gain, *LGA* large for gestational age, *SGA* small for gestational age.

The maternal-infant adverse outcomes were significantly different in the underweight, normal weight, overweight and obese groups. Compared with the other three groups, GDM women in the underweight group more suffered from SGA. The incidence of SGA in the underweight group was 10.54% (214 of 2031), while the incidence of SGA in the obese group was only 3.91% (9 of 230) (Table 3). In contrast, other maternal-infant adverse outcomes, such as LGA, macrosomia and caesarean delivery, mostly occurred in the obese group. A total of 55.22%, 21.14% and 13.04% of GDM women in the obese group suffered from caesarean delivery, LGA or macrosomia, respectively (Table 3). The incidence of prematurity was not significantly different among the underweight, normal weight, overweight and obese groups (Table 3).

Moreover, the GDM women in the underweight, normal weight, overweight and obese groups were further divided into three subgroups based on the recommended GWG ranges by NAM. We found that women in the higher than recommended GWG group had the highest risks of adverse outcomes than the other two subgroups, whereas the lower than recommended GWG group had the lowest risks of adverse outcomes. However, in the underweight group, the equal to recommended GWG group had the lowest risks of adverse outcomes (Table 3).

### Associations of GWG with the total risks of maternal-infant adverse outcomes in each prepregnancy BMI subgroup

**Figure 2:**
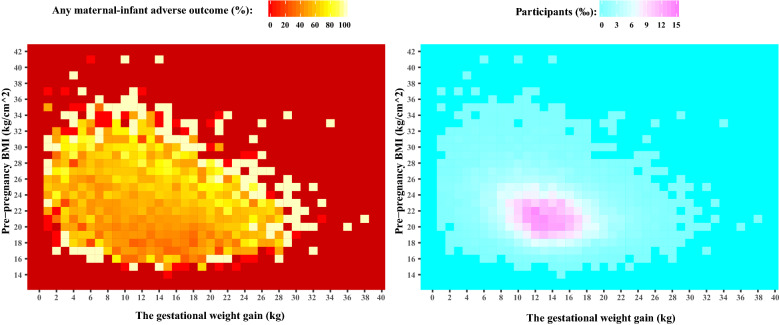
The risk heat map of the maternal-infant adverse outcomes. (**a**) Values represented the risks of any maternal-infant adverse outcomes. (**b**) The percentages of participants for each combination of BMI and GWG.

**Figure 3:**
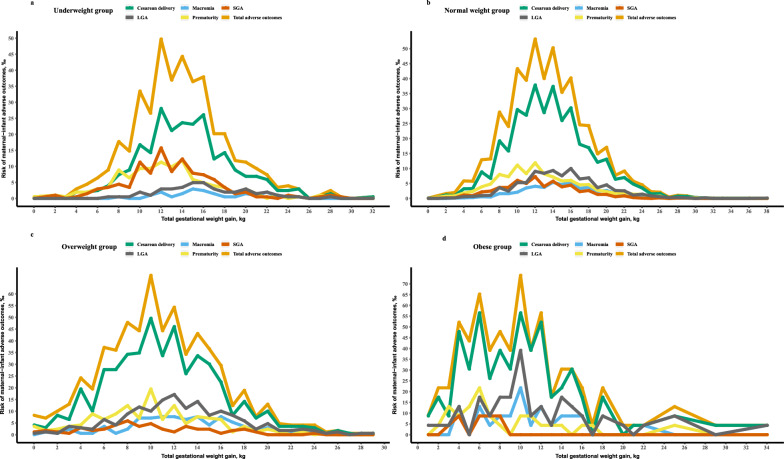
The risks of the maternal-infant adverse outcomes in four pre-pregnancy BMI subgroups. (**a**–**d**) The risks for adverse maternal-infant outcomes in underweight, normal weight, overweight and obesity group were demonstrated respectively. *LGA* large for gestational age, *SGA* small for gestational age.

The absolute risks for any maternal-infant adverse outcome were increased across maternal prepregnancy BMI and were independent of GWG (Fig. 2). The GWG ranges most associated with the maternal-infant outcomes in the underweight, normal weight, overweight and obese groups were determined. The underweight group with a GWG of 4–11 kg had lower risks of maternal-infant adverse outcomes, and women with GWG ranging between 12 and 17 kg had higher risks for adverse outcomes (Fig. 3a). Women in the normal weight group with a GWG of 4–10 kg had lower risks for adverse outcomes, while women with a GWG ranging from 10 to 16 kg had higher risks of adverse outcomes (Fig. 3b). In the overweight group and obese group, women with a GWG of 0–6 kg or GWG of 0–4 kg had lower risks for adverse outcomes, while women with a GWG of 6–11 kg or more than 4 kg had higher risks for adverse outcomes (Fig. 3c,d).

### Associations of maternal prepregnancy BMI and GWG with maternal-infant adverse outcomes

Using logistic regression analysis, we determined the associations of maternal prepregnancy BMI and GWG with each maternal-infant adverse outcome. We showed that the incidences of LGA, macrosomia, caesarean delivery and total adverse outcomes were significantly increased with increasing prepregnancy BMI (*p* < 0.001) (Table 4). In contrast, the risk of SGA was significantly decreased with the increasing prepregnancy BMI (*p* < 0.001) (Table 4). However, the incidence of prematurity was not associated with maternal prepregnancy BMI (Table 4).

**Table 4:** The odd ratios (95% confidence intervals) of maternal-infant adverse outcomes by joint effects of maternal prepregnancy body mass index and gestational weight gain.

Maternal-infant adverse outcomes	Underweight group (n = 2031, BMI < 18.5)	Normal weight group (n = 10,623, 18.5 ≤ BMI < 25.0)	Overweight group (n = 1694, 25.0 ≤ BMI < 30.0)	Obese group (n = 230, BMI ≥ 30.0)	*p*-value
**LGA**					
	0.368 (0.291, 0.466)	1	2.272 (1.952, 2.646)	4.364 (3.132, 6.081)	*p* < 0.001*
Below	0.419 (0.223, 0.787)	0.706 (0.561, 0.889)	0.882 (0.538, 1.446)	1.055(0.364, 3.063)	
Within	1	1	1	1	
Above	1.992 (1.187, 3.342)	1.050 (0.832, 1.325)	0.935 (0.604, 1.448)	1.394(0.518, 3.746)	
*p*-value	*p* = 0.453	*p* < 0.001*	*p* = 0.011*	*p* = 0.085	
**SGA**					
	1.899(1.605, 2.246)	1	0.716 (0.562, 0.913)	0.549 (0.279, 1.082)	*p* < 0.001*
Below	1.103 (0.682, 1.783)	1.031(0.787, 1.351)	1.131 (0.538, 2.377)	1.843(0.156, 21.829)	
Within	1	1	1	1	
Above	1.042 (0.534, 2.031)	0.996 (0.731, 1.357	0.892 (0.402, 1.978)	0.00	
*p*-value	*p* < 0.001*	*p* < 0.001*	*p* = 0.419	p = 0.060	
**Prematurity**					
	0.956	1	0.997	1.175	*p* = 1.00
Below	1.045	0.940	0.821	0.055	
Within	1	1	1	1	
Above	1.120	1.139	0.976	9.848	
*p*-value	*p* = 1.00	*p* = 1.00	*p* = 1.000	*p* = 1.00	
**Macrosomia**					
	0.322 (0.231, 0.447)	1	2.365 (1.943, 2.878)	4.761 (3.164, 7.163)	*p* < 0.001*
Below	1.364 (0.435, 4.277)	0.930 (0.688, 1.257	1.178(0..616, 2.254)	0.648(0.182, 2.313)	
Within	1	1	1	1	
Above	0.640 (0.185, 2.216)	1.010 (0.746, 1.368)	0.756(0.431, 1.326)	1.738(0.708, 4.267)	
*p*-value	*p* = 0.059	*p* < 0.001*	*p* = 0.002*	*p* = 0.321	
**Cesarean delivery**					
	0.528 (0.420, 0.664)	1	1.744 (1.488, 2.044)	2.412 (1.648, 3.532)	*p* < 0.001*
Below	0.845 (0.506, 1.409)	0.931 (0.742, 1.168)	1.074 (0.663, 1.740)	0.621(0.223, 1.731)	
Within	1	1	1	1	
Above	2.205 (1.256, 3.873)	1.210 (0.946, 1.548)	1.123(0.703, 1.794)	0.662(0.284, 1.546)	
*p*-value	*p* = 0.443	*p* < 0.001*	*p* = 0.092	*p* = 0.363	
**Total adverse outcomes**					
	0.780(0.691, 0.880)	1	1.742 (1.546, 1.962)	2.489 (1.857, 3.335)	*p* < 0.001*
Below	1.014 (0.696, 1.476)	0.934 (0.801, 1.090)	1.092(0.761, 1.568)	0.944(0.371, 2.404)	
Within	1	1	1	1	
Above	1.896 (1.170, 3.074)	1.170 (0.988, 1.386)	1.086(0.756, 1.559)	0.645(0.258, 1.612)	
*p*-value	*p* = 0.019*	*p* < 0.001*	*p* = 0.079	*p* = 0.995	

*BMI* body mass index, *GWG* Gestational weight gain.

Moreover, in the underweight group, the incidences of LGA, prematurity, macrosomia and caesarean delivery were not correlated with the GWG. However, the risks of SGA were significantly higher in the subgroup with GWG lower than the NAM recommendation (*p* < 0.001) (Table 4). In contrast, the incidence of total adverse outcomes was significantly higher in the subgroup with GWG higher than the NAM recommendation (*p* < 0.001) (Table 4). The risks of LGA, macrosomia, and caesarean delivery were significantly higher (*p* < 0.001), while the incidence of SGA was significantly lower (*p* < 0.001) in the normal weight group with a GWG higher than the NAM recommendation. The risks of prematurity were not associated with the GWG in the normal weight group (Table 4). In the overweight group, the risks of SGA, prematurity, caesarean delivery and total adverse outcomes were not significantly different among the different GWG subgroups. However, the incidence of LGA was significantly higher in the subgroup above the recommended GWG (*p* < 0.05), while the risk of macrosomia was significantly higher in the subgroup below the recommended GWG (*p* < 0.05) in the overweight group (Table 4). In the obese group, the incidences of LGA, SGA, prematurity, macrosomia, caesarean delivery and total adverse outcomes were not associated with GWG (Table 4).

### Meta-analysis of the associations of maternal-infant adverse outcomes with prepregnancy BMI in three independent cohorts

**Figure 4:**
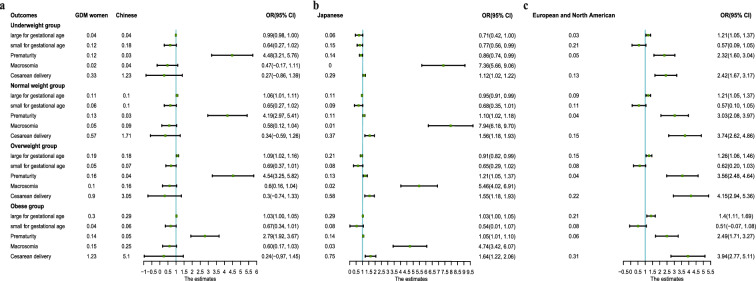
Meta-analysis of the associations of maternal-infant adverse outcomes with pre-pregnancy BMI in three independent cohorts. (**a**) Forest plot showed the different adverse outcomes between women with GDM and normal Chinese women in four pre-pregnancy BMI subgroups. (**b**) The different adverse outcomes between women with GDM and Japanese women in four pre-pregnancy BMI subgroups were compared. (**c**) Compared with Chinese GDM women, the incidences of adverse outcomes in European and North American women were tested.

At last, we determined the maternal-infant adverse outcomes in different prepregnancy BMI subgroups using three independent cohorts, including normal Chinese women^17^, Japanese women^18^, and European and North American women^14^. The normal and GDM Chinese women had no significant differences in the incidences of SGA, caesarean delivery or macrosomia. However, the risks of prematurity from all prepregnancy BMI groups and LGA in the normal weight group and the overweight group among women with GDM were significantly higher than those of normal Chinese women (Fig. 4a). Compared with Japanese women, the incidences of caesarean delivery and macrosomia in the four prepregnancy BMI groups were significantly higher in GDM women. Additionally, in Chinese GDM women, the risks of prematurity in the normal weight group and in the overweight group were significantly higher than those of Japanese women, while the risk of prematurity in the underweight group had the opposite correlation (Fig. 4b). Moreover, in Chinese women with GDM, the risks of LGA, prematurity and caesarean delivery in the four prepregnancy BMI groups were significantly higher than those in European and North American women (Fig. 4c). In contrast, the risks for SGA were lower than those for European and North American women (Fig. 4c).

## Discussion

Considering the ethnic differences between Chinese women and women in Europe or North America, their GWG should be different. Moreover, women who have a better diet and more exercise during pregnancy should have a lower GWG and lower risks of maternal-infant adverse outcomes^16^. In this study, we showed a wider range of GWG among Chinese women with GDM compared with the NAM recommended ranges. Moreover, GWG in the obese group had a wider GWG range than that in the normal weight, overweight and obese groups.

The NAM guidelines and other studies^14,15^ suggested that GWG was a strong predictor of maternal-infant adverse outcomes^19^. Our analysis validated the results that GWG had a negative correlation with maternal-infant adverse outcomes in Chinese women with GDM. Chinese women with GDM in the normal weight, overweight and obese groups who experienced a lower GWG were associated with lower risks of adverse outcomes. The ranges of GWG that were most associated with low risks of maternal-infant adverse outcomes for Chinese women with GDM were 4–11 kg, 4–10 kg, 0–6 kg, and 0–4 kg for the underweight, normal weight, overweight and obese groups, respectively.

Furthermore, the risks of LGA, SGA, prematurity, macrosomia or caesarean delivery had different correlations with GWG. Similar to previous studies^20^, the risks of LGA, macrosomia and caesarean delivery were strongly associated with the increases in prepregnancy BMI and GWG, particularly in the obese group. Previous studies^2,3^ and our analysis suggested that obese women with GDM had a higher incidence of LGA than normal weight women. In contrast, a GWG lower than the NAM recommended among Chinese women with GDM was associated with a higher risk for SGA and prematurity. We also demonstrated that a GWG higher than the NAM recommendation was associated with a lower risk of premature birth, particularly for GDM women with a GWG lower than the NAM recommended in the obese group. Consistent with previous study^21^, pregnant women with a GWG lower than the recommended value were at a higher risk of premature delivery. Moreover, the maternal-infant adverse outcomes in different prepregnancy BMI subgroups were significantly different from those of normal Chinese women, Japanese women, and European and North American women.

As social factors, Chinese women would like to choose caesarean delivery. According to the WHO’s survey, caesarean section rate is 46.2% in China in 2010^22^. However, in last decades, multiple measurements were developed to reduce the caesarean section rate and in this study, only 36% of GDM women were suffered from caesarean delivery. All the caesarean sections were carried out with clear indications and were fully informed the pregnant women. Moreover, we found that, the caesarean section rate was significantly different in the underweight, normal weight, overweight and obese groups. The the caesarean section rate was highest in the obese group. In contrast, women in the underweight group had a relatively low rate of caesarean delivery. Furthermore, caesarean delivery was associated with the prepregnancy BMI and GWG in normal weight group. Our analysis suggested that GDM women with high prepregnancy BMI and GWG more likely required caesarean delivery.

To the best of our knowledge, this is the largest retrospective cohort study to identify the ranges and reveal the associations of GWG and maternal-infant adverse outcomes among Chinese women with GDM. We also used maternal age, prepregnancy BMI and weight, and mode of delivery to adjust for significant potential confounding factors.

However, there were several limitations to this study. First, this was a retrospective cohort study, which may have been biased because of the independent processes of data collection and analysis. Second, we did not analyse the diet and physical activity of the women with GDM during their pregnancy. However, we found that most of the GDM patients in this study were alleviated after dietary intervention and exercise intervention and without further insulin treatment. We had showed that probiotic supplements in diet could alleviate the symptom of GDM in GDM rat model through regulation of gut microbiota and metabolites^23^. Now, we tried our best to study the influence of diet on the treatment of GDM in GDM cohorts. Finally, we only used cohorts from one hospital. Therefore, the collected data and findings may not be representative of Chinese women with GDM. In the future, a prospective cohort study of the ranges of GWG among GDM women from multiple centres in China should be carried out.

## Methods

### Study design and participants

This was a retrospective cohort study in a tertiary public maternity and children’s hospital in China. A total of 17,216 women were diagnosed with GDM from 2013 to 2018 in our hospital. Finally, 14,578 sets of data from 14,334 GDM patients with singleton delivered were used in this study. Among that, 244 GDM patients delivered two times in this hospital. The participants’ information, such as maternal age, occupation, maternal prepregnancy BMI, prepregnancy weight and gestational week, latest weight before childbirth, gestational week at delivery, birth weight of neonate and maternal-infant adverse outcomes, was collected. Women who had more than one singleton pregnancy during the study period were analysed more than once. All participants were diagnosed with GDM according to the diagnostic criteria for GDM defined by the World Health Organization and International Association of Diabetes and Pregnancy Study Groups. When one or more of the following test results are recorded during antenatal visits between 24 and 28 gestational weeks or at any other time during the course of pregnancy: (1) Fasting plasma glucose levels 5.1–6.9 mmol/L (92–125 mg/dL). (2) One-hour oral glucose tolerance test (OGTT) values were greater than or equal to 10.0 mmol/L (180 mg/dL) after a 75 g oral glucose load. (3) Two-hour OGTT values between 8.5 and 11.0 mmol/L (153–199 mg/dL) after a 75 g oral glucose load.

### Classification of the GDM women

Due to the GWG criteria of Chinese pregnant women refer to NAM recommendation, which recommended different GWG range according to four prepregnancy BMI groups, so all women were stratified into four weight groups based on prepregnancy BMI: underweight (BMI < 18.5 kg/m^2^), normal weight (18.5 kg/m^2^ ≤ BMI < 25.0 kg/m^2^), overweight (25.0 kg/m^2^ ≤ BMI < 30.0 kg/m^2^) and obese (BMI ≥ 30.0 kg/m^2^). GDM women in each group were further divided into three subgroups based on the recommended GWG ranges by NAM: lower, equal or higher than the recommended range subgroups. GWG was defined as the weight difference between prepregnancy and just before delivery. The prepregnancy weight was either measured at the first antenatal visits before 12 gestational weeks or self-reported by the participants. Just before delivery, their weight was measured in the obstetrical ward. The prepregnancy BMI was calculated by dividing the prepregnancy weight by the square height (m^2^) of the GDM women. BMI was also calculated by dividing the square height (m^2^) of the antenatal visits by the pregnancy weight of the participants.

### Maternal-infants adverse outcomes

The maternal-infant adverse outcomes included large for gestational age (LGA), small for gestational age (SGA), prematurity, macrosomia and caesarean delivery. Gestational age-adjusted standard deviation for birth weight was calculated using a China reference chart^24^. SGA and LGA were defined as gestational age-adjusted birth weights less than the 10^th^ percentile and greater than the 90th percentile, respectively. Prematurity was defined as birth at less than 37 weeks of gestation^25^. Macrosomia was defined as neonates with birth weights over 4000 g.

### Comparison of maternal-infant adverse outcomes of this study with other cohorts

Searching prepregnancy BMI and maternal-infant adverse outcomes in PubMed, we selected three independent cohorts: normal Chinese women^17^, Japanese women^18^, and European and North American women^14^, for the comparison analysis. The adverse outcomes in each cohort were compared with women with GDM. The 95% CIs of the ORs for adverse outcomes were calculated based on a computational formula. Continuous data were compared using one-sample t-tests. The *p* < 0.05 was considered significantly different.

### Statistical analysis

Python software (version 3.7.0) was used to process the data. R software (version 3.5.1) was used for data analysis and plotting, and SPSS software (version 25.0) was also used for data analysis. If the data followed a normal distribution, a t-test or post hoc test was performed to analyse the data. Otherwise, nonparametric tests were conducted. The data are presented as the mean ± standard deviation (mean ± SD). The risks for maternal-infant adverse outcomes were categorized as yes or no, and logistic regression models were used to estimate odds ratios [95% confidence intervals (95% CI)] of maternal prepregnancy BMI (four groups) and GWG on the risks of maternal-infant adverse outcomes. Variables including maternal age, occupation, prepregnancy BMI, gestational BMI gain and GWG were entered into the logistic regression models by Backwards: Wald. A *p value* < 0.05 indicates a significant difference.

### Ethics statement

This study was carried out in accordance with the guidelines and approved by the Ethical Committee of Fujian Maternity and Child Health Hospital (Ref.: 2019161). Informed consent was obtained from all the participants.
